# Construction of drought stress regulation networks in potato based on SMRT and RNA sequencing data

**DOI:** 10.1186/s12870-022-03758-8

**Published:** 2022-08-01

**Authors:** Hongju Jian, Haonan Sun, Rongrong Liu, Wenzhe Zhang, Lina Shang, Jichun Wang, Vadim Khassanov, Dianqiu Lyu

**Affiliations:** 1 College of Agronomy and Biotechnology, Southwest University, Chongqing, 400715 China; 2 State Cultivation Base of Crop Stress Biology for Southern Mountainous Land of Southwest University, Chongqing, 400715 China; 3 Chongqing Key Laboratory of Biology and Genetic Breeding for Tuber and Root Crops, Chongqing, 400715 China; 4 S. Seifullin Kazakh Agrotechnical University, Zhenis Avenue, 010011 Astana, Republic of Kazakhstan

**Keywords:** *Solanum tuberosum*, Drought stress, SMRT, Alternative splicing, LncRNAs, Alternative polyadenylation

## Abstract

**Background:**

Potato (*Solanum tuberosum*) is the fourth most important food crop in the world and plays an important role in food security. Drought stress has a significantly negative impact on potato growth and production. There are several publications involved drought stress in potato, this research contributes to enrich the knowledge.

**Results:**

In this study, next-generation sequencing (NGS) and single-molecule real-time (SMRT) sequencing technology were used to study the transcription profiles in potato in response to 20%PEG6000 simulates drought stress. The leaves of the variety “Désirée” from in vitro plantlets after drought stress at six time points from 0 to 48 hours were used to perform NGS and SMRT sequencing. According to the sequencing data, a total of 12,798 differentially expressed genes (DEGs) were identified in six time points. The real-time (RT)-PCR results are significantly correlated with the sequencing data, confirming the accuracy of the sequencing data. Gene ontology and KEGG analysis show that these DEGs participate in response to drought stress through galactose metabolism, fatty acid metabolism, plant-pathogen interaction, glutathione metabolism and other pathways. Through the analysis of alternative splicing of 66,888 transcripts, the functional pathways of these transcripts were enriched, and 51,098 transcripts were newly discovered from alternative splicing events and 47,994 transcripts were functionally annotated. Moreover, 3445 lncRNAs were predicted and enrichment analysis of corresponding target genes was also performed. Additionally, Alternative polyadenylation was analyzed by TADIS, and 26,153 poly (A) sites from 13,010 genes were detected in the Iso-Seq data.

**Conclusion:**

Our research greatly enhanced potato drought-induced gene annotations and provides transcriptome-wide insights into the molecular basis of potato drought resistance.

**Supplementary Information:**

The online version contains supplementary material available at 10.1186/s12870-022-03758-8.

## Introduction

Drought is the most important environmental factor affecting crop yield. Studies have shown that drought can cause up to 50% of crop yield loss. As global warming intensifies the effect, frequency and range of drought, results in more serious yield loss [23]. Therefore, development of breeding drought tolerant materials is an important goal of current breeding. Although lots of work on drought stress have been conducted in model plants, the molecular mechanism of drought response in potato, the fourth largest food crop, still needs to be further explored. The yield of potato is far from its physiological potential. Due to the drought-sensitivity of potato varieties, the yield is greatly reduced due to the lack of irrigation facilities or arid areas in the world. Drought stress leads to biochemical, molecular, physiological and morphological changes and significantly affects plant growth and yield [59]. Materials with strong drought tolerance can reduce the impact of water shortage on plants. Therefore, screening and utilizing candidate drought stress response genes can stabilize potato yield in water-shortage area.

Plants have evolved very complex regulatory mechanisms to cope with the adverse environments [6, 51, 88]. Plants resist drought stress by reducing water loss (such as decreased leaf area, reduced stomata number and its conductance) and increasing water uptake (enhanced root formation, increased leaf thickness, and leaf rolling) in morphological aspect [65]. Many studies have been conducted to explore the impact of drought stress on physiological, molecular, and biochemical levels in various crops [37]. Aliche et al. [4] revealed the mechanism of potato carbon partition under drought stress. The development of new knowledge and techniques on omics high-throughput approaches is crucial to potato drought resistance [11], Obidiegwu et al. [53] presented an overview of drought stress and adaptive responses in potato, indicated that omics analysis was future research directions. Comparing transcript levels differences between drought-tolerant and sensitive cultivars can effectively understand the drought response mechanism of potato [67] [12].) identify key mechanisms and processes underlying single and combined abiotic stress tolerance by comparative analysis of tolerant and susceptible cultivars. Gervais et al. [20] has revealed that leaf protein content, instantaneous WUE, stomatal conductance, and transpiration rates can be used as the screening criteria for selecting the drought-tolerant potato genotypes. There have been many reports on the rapid identification of the molecular mechanisms of drought response at the whole-genome level using next-generation sequencing (NGS) platforms or gene microarrays in different species. Based on these data, key genes, proteins or pathways were identified. Genes encoding proteins which are involved in water transport (aquaporin) [2, 3], scavenging of free oxygen radicals (superoxide dismutase, catalase, and peroxidase), maintaining cellular membrane integrity (proline, mannitol, glycine, and betaine), and protecting macromolecules (chaperones and late embryogenesis abundant proteins) [21, 63] directly protects plants against drought stress. The other genes involved in signal perception or transduction may also play critical roles in response to drought stress. Key transcription factors (TFs) such as MYB, WRKY, bZIPs, kinases such as SnRK2 and small molecules such as abscisic acid (ABA) have been functionally proved to play key roles in plant drought tolerance (Shinozaki, Yamaguchi -[21, 64]). Some key TFs such as ABA-dependent MYC/MYB, AREB/ABF (ABA-responsive element binding/ABA-binding factor), DREB (ABA-independent dehydration-responsive element-binding proteins), CRT/DRE (C-repeat/drought-responsive element) have been proved in many crops in response to drought stress [28, 62, 82].

The *S. tuberosum* group Phureja clone DM1–3516 R 44 (doubled monoploid) and the *S. tuberosum* group Tuberosum RH89–039-16 (heterozygous diploid) have been sequenced and released via the Potato Genome Sequence Consortium (PGSC) in 2011 [81]. There are new and improved sequences done for both diploid and tetraploid potato [55, 86]. These several genome sequences have been released and become in great genomic resources available, and provides a very good genomic reference information for transcriptome sequencing [19, 22, 46, 47]. For drought stress in potato, several studies have been conducted using RNA-Seq [10, 49, 66, 85]. In addition, Petek et al. compiled available and newly generated RNA-Seq datasets for three tetraploid potato genotypes (cultivar Désirée, cultivar Rywal, and breeding clone PW363) with diverse breeding pedigrees [54]. Some studies on genomic assembly of tetraploid potato cultivar have also been reported recently [27, 69]. In this study, we assembled RNA-Seq datasets for Désirée in response drought stress.

Although NGS technology has the advantages of high throughput, high sensitivity, high accuracy and low cost, its main disadvantage is that the reading length is short, and it is difficult to ensure the accuracy of the reconstructed transcript in the assembly process. This presents a serious challenge to eukaryotic transcriptome sequencing with many homologous genes [68]. In order to break the barrier, the third generation sequencing technology came into being. The third generation sequencing technology is a single molecule sequencing technology. In the process of sequencing, each RNA molecule is directly sequenced without PCR amplification. It has the characteristics of super long reading length and can cover the complete transcripts. The single molecular real time (SMRT) sequencing technology introduced by Pacific Bioscience (pacbio) was most widely used [24]. Through this sequencing technology, information such as variable splicing, fusion gene, gene family and lncRNA can be accurately identified, which is widely used in genome research, transcriptome sequencing and epigenetic analysis [57]. However, the main problem in SMRT is the high error rate (mainly reflected in insertion or deletion errors) [57]. In order to solve this problem, the NGS with high quality and high coverage sequencing data was used to correct SMRT data, so “SMRT + NGS” sequencing joint analysis is more and more widely used [8, 83, 84].

In previous studies on potato drought stress transcriptome sequencing, although many differentially expressed genes were identified and screened, clustered and functionally annotated, their analysis was not comprehensive and detailed [16, 22, 85]. In our study, the leaves of the variety “Désirée” at the young plants stage after drought stress at six time points from 0 to 48 hours were used to perform NGS and SMRT sequencing. Our research will provide a valuable supplement to the potato genome and a broader regulatory mechanism in response to drought stress in potato.

## Materials and methods

### Plant materials and drought stress treatments

A drought tolerant tetraploid potato “Désirée ”[45, 49], provided by Southwest University, was used in this study. In 2 weeks old vitro plantlets of about 10 cm were transferred and stablished into hydroponic solution (1/4 Hoagland nutrient solution) for 1 week at 22/18 °C (16 h light / 8 h dark), then the materials were divided into two parts, one for control and the other for drought stress treatment to simulate the drought stress as reported by different authors, which were treated with 20% polyethylene glycol (PEG)-6000 [25, 48, 76]. The aboveground tissues were taken at 0, 1, 3, 6, 12, 24 and 48 hours after drought stress treatment and immediately frozen at least 30 min in liquid nitrogen and kept at − 80 °C until use. The samples were collected for three biological replicates. The collected samples were sent to Biomarker for full-field transcriptome sequencing and second-generation transcriptome sequencing, including sample detection, library construction and sequencing.

### Library construction for Illumina and PacBio sequencing

Total RNA of all samples was extracted using EZ-10 DNAaway RNA Mini-Preps Kit (Sangon Biotech, Shanghai, China) and the quality of total RNAs was evaluated using an Agilent 2100 Bio-analyzer (SA Pathology, Adelaide, SA, Australia). High-quality RNAs (RIN > 8.0) were used for the following cDNA synthesis and library construction. For Illumina RNA sequencing, 1.0 μg RNA per sample was used for cDNA synthesis with the NEBNext® Ultra™ II RNA Library Prep Kit for Illumina (NEB, USA) and sent to Biomarker Technologies Corporation (Beijing, China) for Illumina sequencing. For SMRT sequencing, equal amount of the total RNA of all samples were pooled together as the template for cDNA synthesis with the SMARTer PCR cDNA Synthesis Kit (Clontech, USA) in Biomarker Technologies Corporation (Beijing, China).

### Analysis of the Illumina and SMRT sequencing data

The raw Illumina data was filtered to generate clean reads using FastQC tool (http://www.bioinformatics.babraham.ac.uk/projects /fastqc/) (v0.11.5) and remove the adaptor contamination and low-quality reads using Trimmomatic (v0.35) [7]. The high-quality clean reads were then mapped to the reference genome database (http://solanaceae.plantbiology.msu.edu/rh_potato_download.shtml) using HISAT2 version 2.1.0 and the default parameters [33]. For SMRT sequencing data, the circular consensus sequences (CCS) were generated from the PacBio Iso-Seq raw reads with smrtanalysis_2.3.0 (http://www.pacb.com/) and then processed into error corrected reads of insert (ROIs) with a minimum full pass of > 3 and minimum accuracy of > 0.9. These ROIs were classified as full-length, non-chemiric (FLNC) transcripts using smrtanalysis_2.3.0 based on the presence and location of the 5′ and 3′ cDNA primers and poly (A) tail. All full-length reads were aligned to the *S. tuberosum* group Tuberosum RH89–039-16 reference genome (http://solanaceae.plantbiology.msu.edu/rh_potato_download.shtml) using GMAP software [78]. Finally, all mapped reads were collapsed using a Python script in the ToFU package (https://github.com/PacificBiosciences/cDNA_primer/) with min-coverage = 85% and min-identity = 90%. All raw data was deposited in the National Center for Biotechnology Information (NCBI) sequence read archive with accession no. PRJNA728834.

### Gene functional annotation, enrichment analysis, and weighted gene co-expression network (WGCNA) analysis

Gene functions was predicted using the following databases: NR (NCBI non-redundant protein sequences), Pfam (protein family), Swiss-Prot, KEGG (Kyoto Encyclopedia of Genes and Genomes), and GO (gene ontology). The GOseq package in R (version 3.4.3) and the KOBAS software were used to perform the GO and KEGG pathway enrichment, respectively. The gene expression levels were calculated using FPKM (fragments per kilobase of exon per million fragments mapped). Genes with FPKM values > 1 in each sample were used for WGCNA analysis using the R package WGCNA (v1.42).

### Differential expression analysis and candidate gene determination

Differentially expressed genes (DEGs) were analyzed by DESeq and detected with the parameter FDR < 0.01 & |Log2Ratio| > 1. The plant transcription factors were predicted by iTAK software, and the differentially expressed transcription factors were screened by mapping to the DEGs database and the genes related to plant hormones in DEGs were screened based on Gene Ontology (GO) functional annotation. Twenty-nine DEGs were randomly selected and verified using the real-time (RT)-PCR to detect the accuracy of transcriptome data (Supplementary Table S1). Toolbox for Biologists v1.087 [9] was used to construct the heatmap.

### Identification of lncRNAs and AS events

In order to analyze the coding potential in the transcripts, four of the most widely used computational approaches including coding potential calculator (CPC) (Li, [85]), coding-non-coding Index (CNCI) [5], coding potential assessment tool (CPAT) [13], and pfam protein structure domain analysis were combined. Transcripts were regarded as lncRNA candidates using the parameter: lengths > 200 nt & > 2 exons.

To identify the AS (Alternative splicing) events, the ASTALAVISTA algorithm [17] based on the assembled GTF file from the PacBio Iso-Seq and Illumina data was conducted and five types of AS events including IR (Intron retention), ES (Exon skipping), A5 (Alternative 5′ splice site), A3 (Alternative 3′ splice site) and MEE (Mutually exclusive exon) were classified with the default settings.

### Isoform identification and reverse transcription (RT)-PCR

To distinguish AS events, the reverse transcription (RT)-PCR was performed. Briefly, the RNA used for Iso-Seq was isolated using EZ-10 DNAaway RNA Mini-Preps Kit (Sangon, https://www.sangon.com) and synthesized to complementary DNA using the Hifair® II 1st Strand cDNA Synthesis SuperMix for qPCR gDNA digester plus (Yeasen). Isoform-specific primers were designed using Vector NTI software (Supplementary Table S1). PCR amplification was performed using 2 × Taq PCR Master Mix (K1034, https://www.apexbt.com/) and the PCR products were showed in agarose gel stained with GoodView™ Nucleic Acid Stain (Apr-13-2021, HGV-II, https://www.sbsbio.com).

BlazeTaq™ SYBR® Green qPCR Mix 2.0 (GeneCopoeia, USA) was used for RT-qPCR reactions on a CFX96 Real-time System (BIO-RAD, USA). The amplification program was briefly described in previous study [73]. To ensure the precise and reproducible results, each sample was replicated three times and Ef1α in potato were used for each sample as an endogenous control (Supplementary Table S1). The formula F = 2 ^–ΔΔCt^ was used to calculate the relative expression levels of selected genes.

### Alternative polyadenylation analysis

Alternative splicing and alternative polyadenylation (APA) of pre-mRNAs greatly contribute to transcriptome diversity, coding capacity of a genome and gene regulatory mechanisms in eukaryotes. TADIS pipeline (Transcriptome Analysis Pipeline for Isoform Sequencing) was used to further analyze the full-length non-chimeric sequence (FLNC) to identify APA [1] and MEME analysis was also performed to identify poly (A) sequence signals in our data.

## Results

### Illumina and PacBio sequencing data analysis

**Fig. 1:**
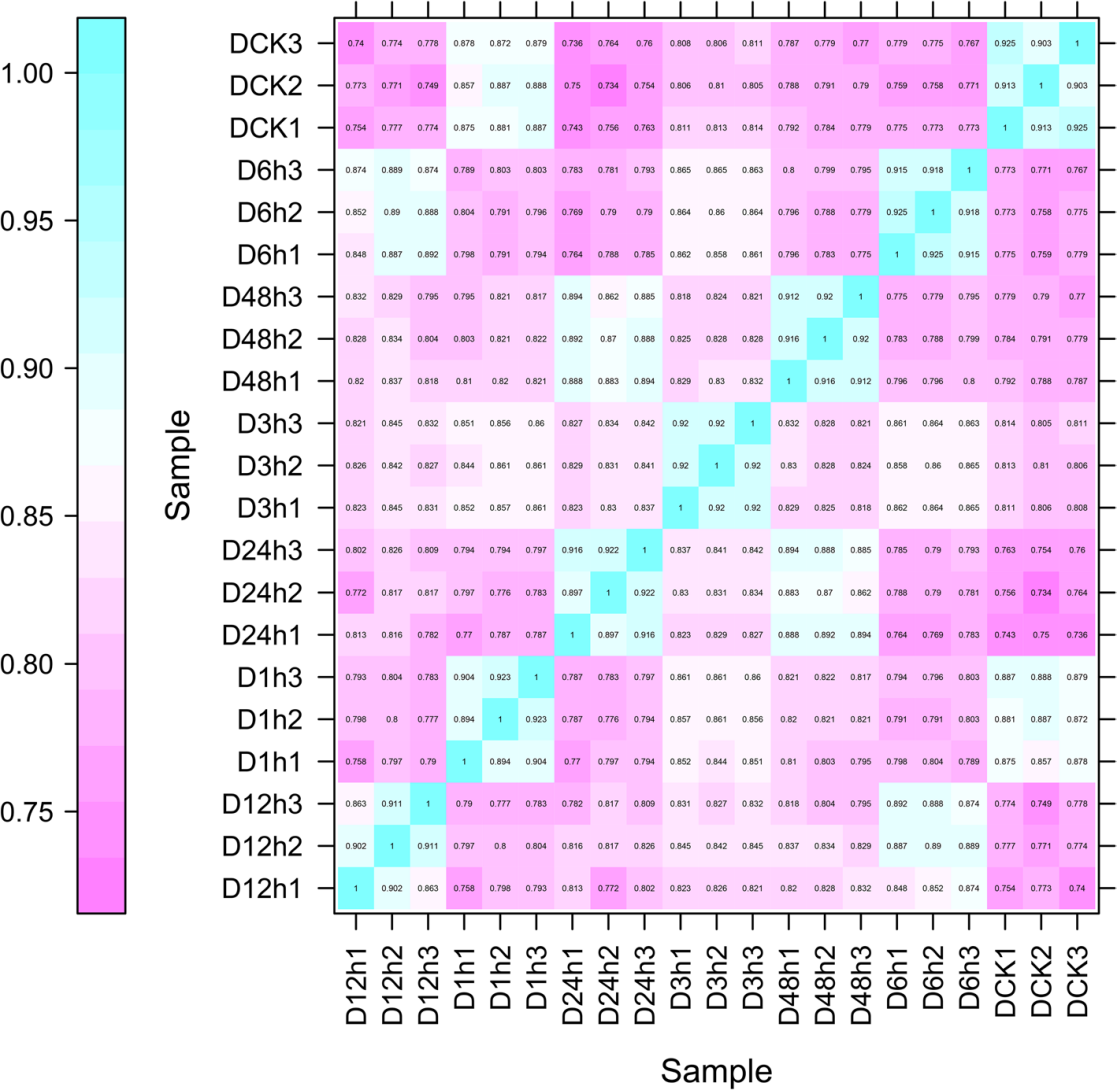
Pearson correlation evaluation among 21 samples. DCK represents control groups; D1h-D48h represents drought treatment after 1 h, 3 h, 6 h, 12 h, 24 h and 48 h. Each sample has three biological replicates

**Fig. 2:**
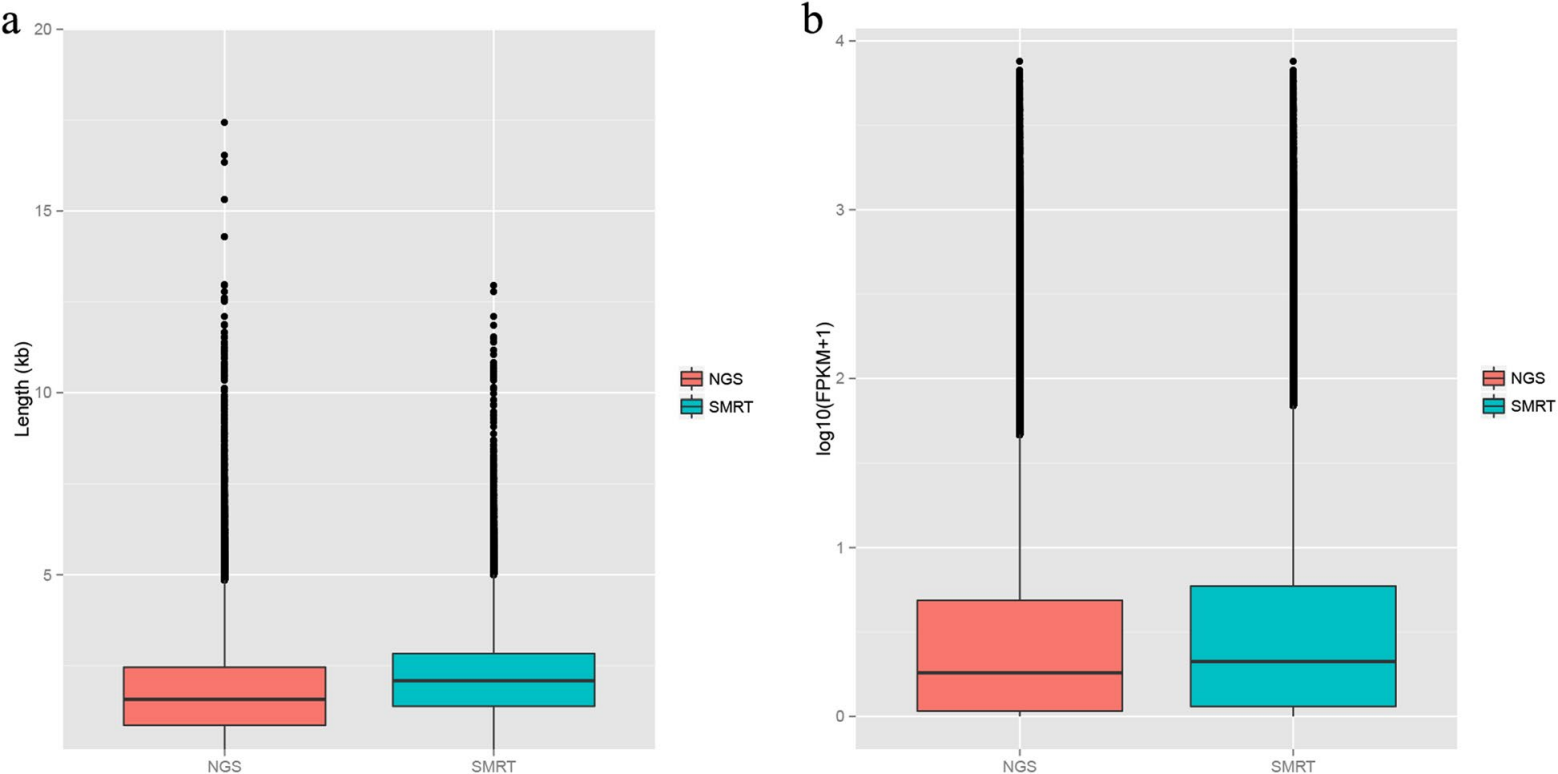
Comparisons between NGS and SMRT. **a**. The difference between NGS and SMRT sequencing length. **b**. The difference in FPKM of NGS and SMRT sequencing

To construct a comprehensive regulation network in response to drought stress, control and treated samples were processed through SMRT sequencing on the PacBio platform and next-generation sequencing (NGS) on the Illumina Hi-Seq platform. High-quality RNA was extracted from above tissues of “Désirée” after PEG-6000 treatments (0 h, 1 h, 3 h, 6 h, 12 h, 24 h and 48 h) for Illumina Hi-Seq and pool together for construction of Iso-Seq library. After removing low-quality reads and adapter sequences, 38,347,790-46,349,352 reads were obtained and more than 80% sequences were mapped to the reference genome (Table 1). The Pearson’s correlation coefficient showed that all correlation values between the three replicates ranged from 0.826 to 0.925, indicating a perfect positive correlation and that the sequencing results could be used for further analysis (Fig. 1). For Iso-Seq, the PacBio platform generated 33.24 Gb clean data. Among these data, 464,332 circular consensus (CCS) reads were obtained using Iso-seq pipeline with min full pass > 3 and min predicted accuracy > 0.9. Then, 375,387 full length non chimeric (FLNC) sequences were determined by searching for the polyA tail signal and the 5′ and 3′ cDNA primers and 121,434 high-quality consensus sequences were obtained by clustering the FLNC sequences. SMRT sequencing can offer better transcript integrity (up to 50 kb in length) than NGS and has greatly contributed in maize [74], populous [8] oilseed rape [84] and sorghum [1, 44], but no literature has been reported in potato. Significantly longer isoforms were detected by SMRT than by NGS assembly, demonstrating better isoform integrity (Fig. 2a). In addition, the overall expression levels of transcripts detected by SMRT were higher than those detected by NGS, indicating better quantification accuracy (Fig. 2b).

**Table 1:** Summary of Illumina transcriptome sequencing data mapping on potato reference genome

Sample	Total Reads	Mapped reads	Uniquely mapped reads	Multiple mapped reads
DCK1	46,126,668	81.98%	62.71%	19.28%
DCK2	39,174,602	81.81%	62.61%	19.20%
DCK3	44,991,258	82.36%	62.88%	19.48%
D1h1	45,228,120	90.92%	70.45%	20.47%
D1h2	40,548,794	81.34%	62.37%	18.97%
D1h3	42,450,306	82.02%	62.97%	19.05%
D3h1	40,378,280	81.80%	63.29%	18.51%
D3h2	41,469,874	81.31%	62.58%	18.72%
D3h3	44,184,948	81.83%	62.87%	18.96%
D6h1	42,527,962	81.93%	62.62%	19.31%
D6h2	43,485,914	82.19%	63.05%	19.14%
D6h3	42,232,976	81.51%	62.45%	19.07%
D12h1	38,347,790	80.85%	61.85%	19.01%
D12h2	42,119,072	81.81%	62.77%	19.04%
D12h3	43,073,848	81.43%	62.03%	19.40%
D24h1	43,781,704	81.35%	62.91%	18.44%
D24h2	46,349,352	83.75%	64.46%	19.30%
D24h3	42,890,028	81.76%	63.05%	18.71%
D48h1	39,744,764	81.57%	62.84%	18.73%
D48h2	43,222,312	81.47%	62.85%	18.62%
D48h3	43,527,026	81.67%	62.90%	18.77%

### Analysis of gene expression and transcriptomic changes in response to drought treatment

**Fig. 3:**
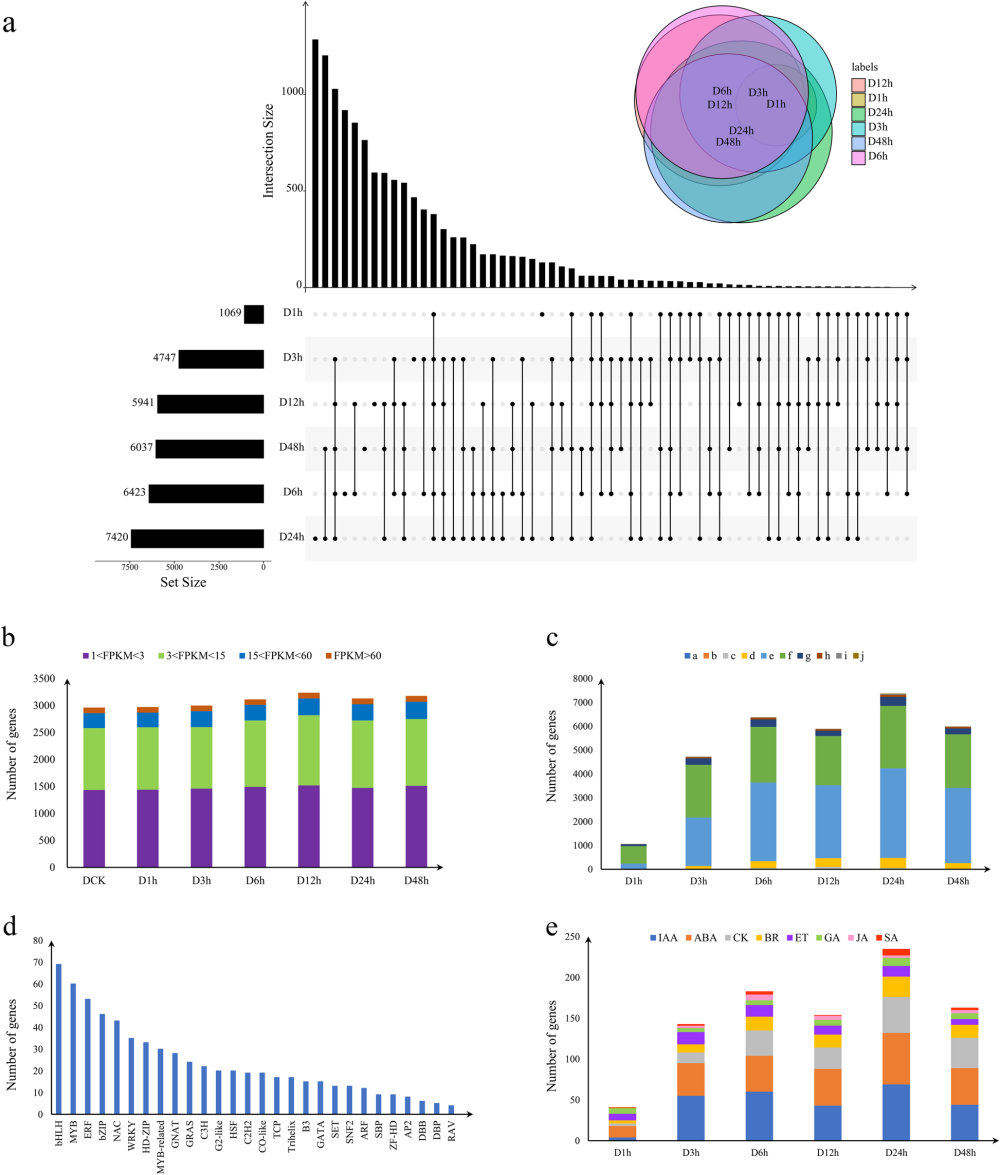
DEGs expression profiles of six samples treated for different periods of drought treatment. **a**. Venn Diagram of genes detected in the six samples. **b**. The number of DEG genes per treatment at different FPKM ranges. **c**. Fold changes in the DEGs identified after drought treatment. [(a) Number of DEGs with a log2 fold change ≤ − 9; (b) number of DEGs with − 9 < log2 fold change ≤ − 7; (c) number of DEGs with − 7 < log2 fold change ≤ − 5; (d) number of DEGs with − 5 < log2 fold change ≤ − 3; (e) number of DEGs with − 3 < log2 fold change ≤ − 1; (f) number of DEGs with 1 < log2 fold change ≤3; (g) number of DEGs with 3 < log2 fold change ≤5; (h) number of DEGs with 5 < log2 fold change ≤7; (i) number of DEGs with 7 < log2 fold change ≤9; (j) number of DEGs with log2 fold change >9]. **d.** The Number of DEGs encoding TF in different families identified. **e.** The number of genes encoding hormones identified in the DEGs. [ABA: abscisic acid; IAA: auxin; BR: brassinosteroids; CK: cytokinin; GA: gibberellin; JA: jasmonic acid; SA: Salicylic acid]

**Fig. 4:**
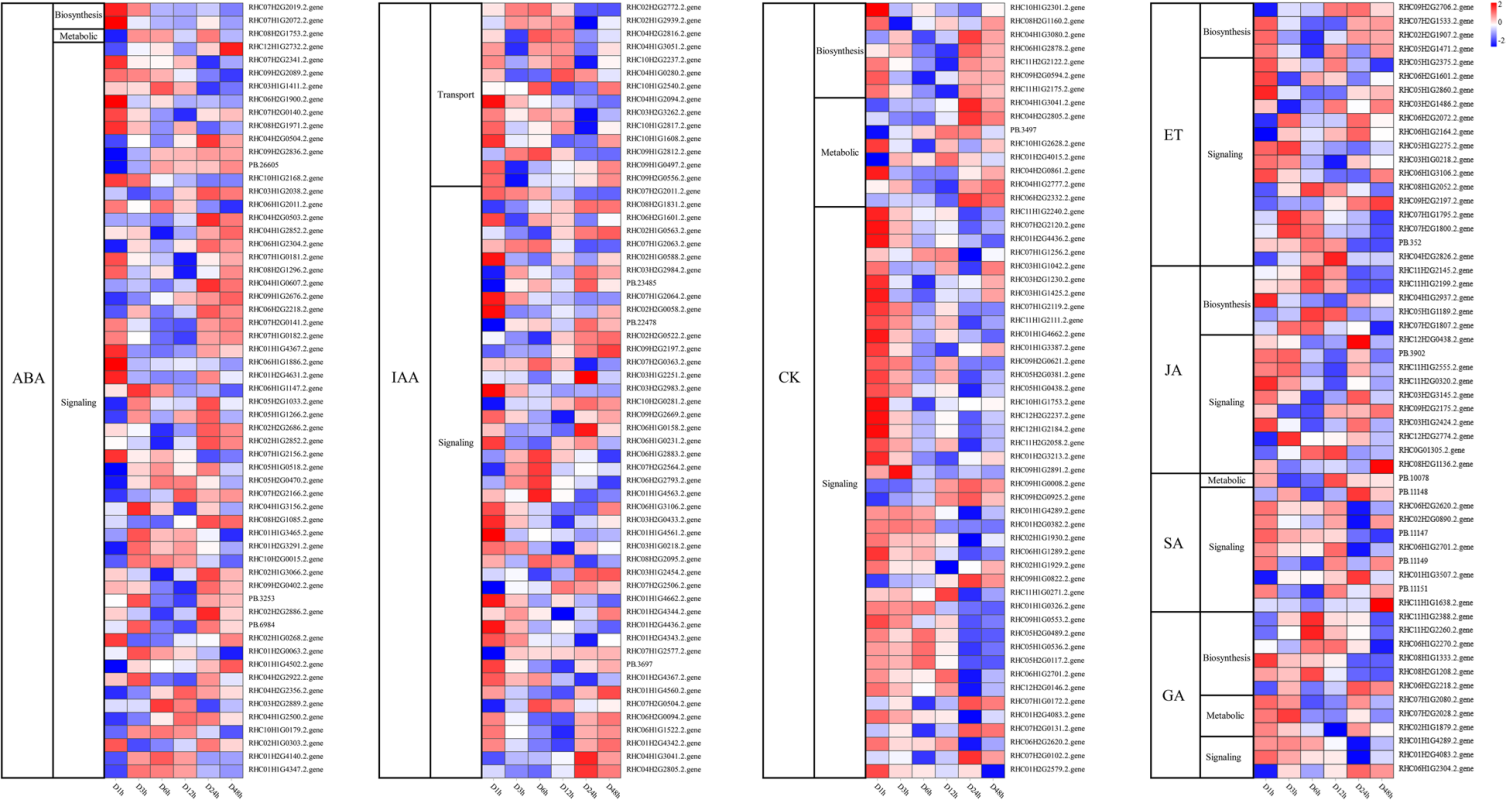
Heat maps of DEGs associated with hormone metabolism and signal transduction in potato. Expression of genes involved in ABA: abscisic acid, IAA: auxin, CK: cytokinin, GA: gibberellin, JA: jasmonic acid, SA: Salicylic acid metabolism and signaling after drought treatment

DESeq was used for differential expression analysis between control versus treatments. False Discovery Rate (FDR) was used as statistical parameter for differential expression gene screening. Fold Change≥2 and FDR < 0.01 were used as screening criteria. In total, 31,637 DEGs were detected between control versus treatments, and the number of DEGs in D24h sample was the largest. (Fig. 3a and Supplementary Table S2). Moreover, almost all of DEGs in the libraries showed moderate expression levels with only a small percentage of the genes expressed in high levels (Fig. 3b and Supplementary Table S3). More than 88% of the DEGs had a − 3<fold change ≤3. Transcription factors (TFs) play important roles in plant abiotic stress responses (Fig. 3c) [29]. To further analyze the function of the DEGs, we used iTAK software to predict transcription factors in DEGs. In total, 664 DEGs belonging to 28 TF families were identified. The TF family members were unevenly distributed. Seven TF families, bHLH (69), MYB (60), ERF (53), bZIP (46), NAC (43) WRKY (35) and HD-ZIP (33) accounted for more than 51% (339/664) of all TFs (Fig. 3d). In total, 919 DEGs belonging to eight classifications of plant hormones, and ABA, IAA and CK-related genes accounted for the majority, accounting for about 94% of all hormone-related genes (Fig. 3e). To combine the hormone profiling data with the expression profiles of genes involved in hormone metabolism and signaling, we analyzed 231 genes involved in plant hormone metabolism and visualized them as heat maps after functional classification (Fig. 4). Heat map results showed that DEGs were most involved in plant hormone signaling.

### Gene profiling in response to drought stress in potato

**Fig. 5:**
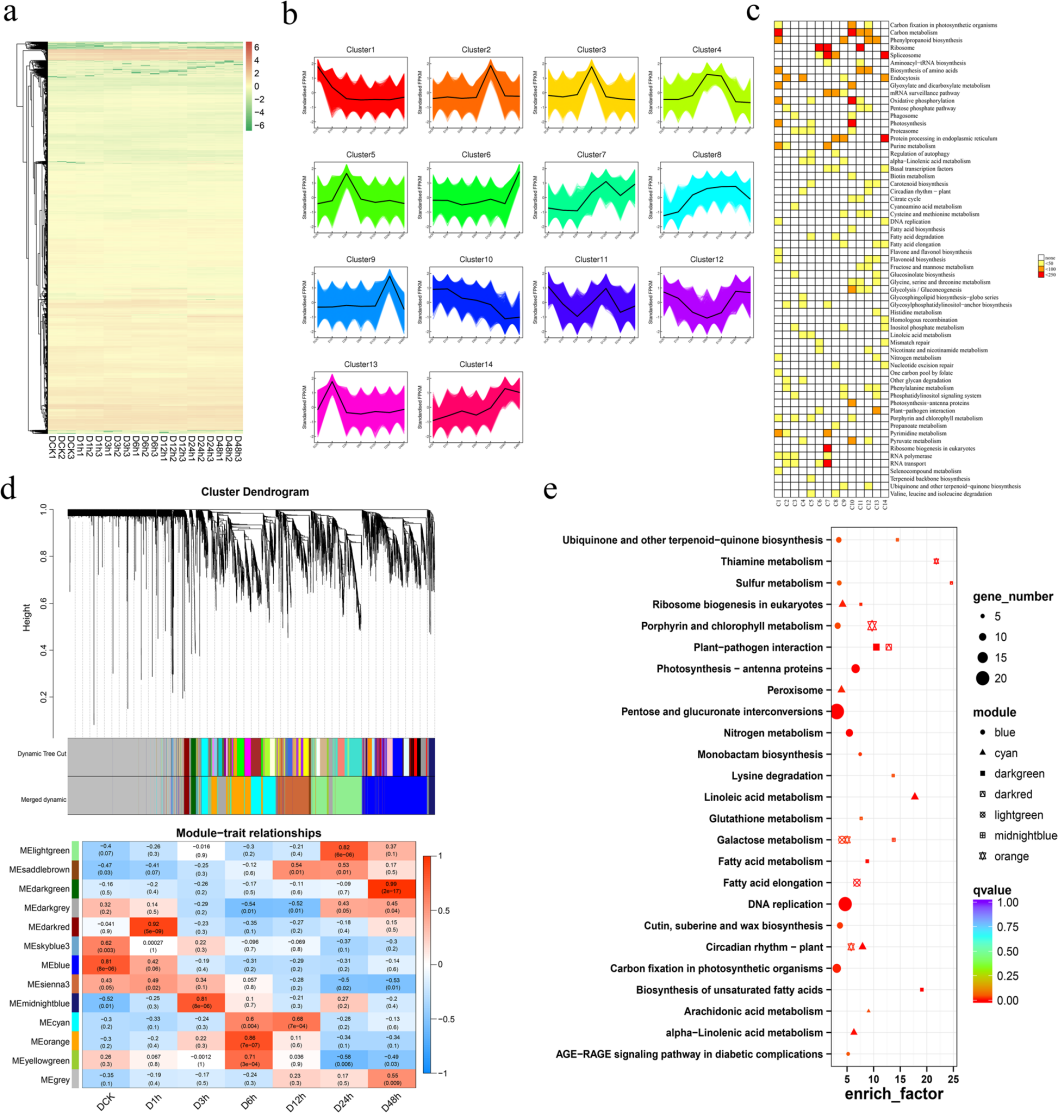
Global patterns of differentially expressed genes (DEGs) under drought stress. **a**. Hierarchical cluster analysis of DEGs. **b**. K-means clustering (C1–C14) showing transcriptome expression profiles. **c**. Gene Ontology enrichment among the 14 clusters. Yellow to red, significant enrichment; white, none. **d**. Weighted gene co-expression network (WGCNA) analysis of DEGs. **e**. Expression levels of genes among the modules most significantly correlated with drought-treated samples

**Fig. 6:**
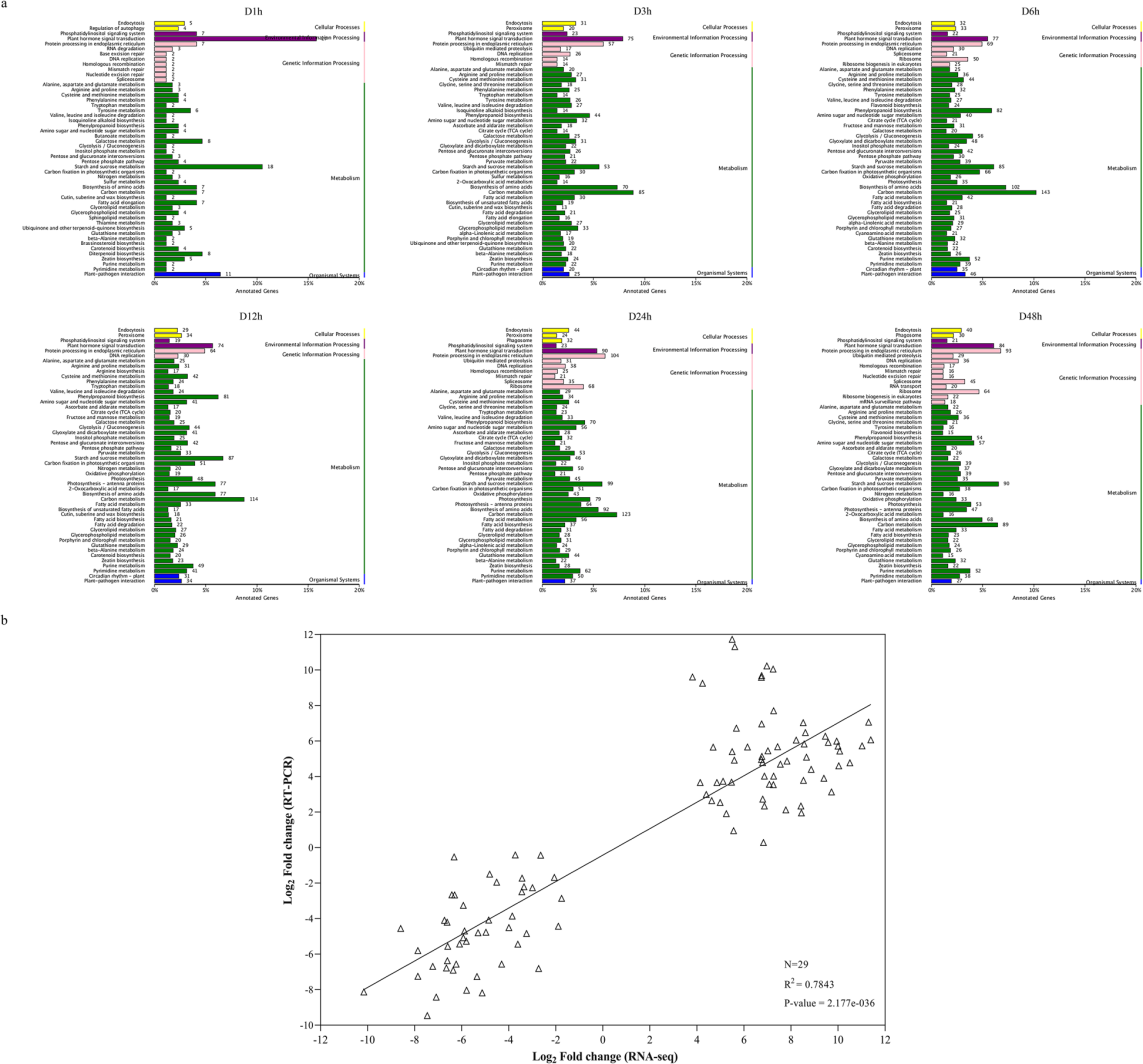
KEGG classification of differentially expressed genes in drought-treated samples and RT-PCR verification of several candidate genes. **a**. KEGG classification of differentially expressed genes in six drought-treated samples (D1h, D3h, D6h, D12h, D24h and D48h) [30–32]. **b**. Correlation evaluation between real-time (RT) PCR and RNA-seq

To explore drought stress response mechanisms in potato, the expression patterns were calculated (Fig. 5a). The hierarchical cluster analysis was done and then the K means algorithm used for 98,624 genes. Fourteen clusters were obtained and 63 pathways were enriched, such as carbon metabolism, carbon fixation in photosynthetic organisms, porphyrin and chlorophyll metabolism, protein processing in endoplasmic reticulum and oxidative phosphorylation (Fig. 5b and c). Furthermore, WGCNA was also performed to screen genes response to drought stress by detecting molecular modules. Thirteen modules were generated and more than one module was significantly correlated with each sample. Blue, darkred, midnightblue, orange, cyan, lightgreen, and darkgreen were significantly correlated with DCK, D1h, D3h, D6h, D12h, D24h and D48h, respectively (Fig. 5d). Then, KEGG enrichment analysis was conducted to explore the enriched pathways in each module correlated to each sample. Galactose metabolism (ko00052) were enriched in more than two treated samples. Plant-pathogen interaction (ko04626) and glutathione metabolism (ko00480) were enriched in early stages after drought stress (Fig. 5e and Fig. S1). In addition, through the comparative analysis of the control group and the other six samples processed in different periods, a total of 12,798 differentially expressed genes after deduplication were found (Fig. S2 and Supplementary Table S4). The functional enrichment of these DEGs during the six drought treatment period was conducted (Fig. 6a) and 29 DEGs were selected to confirm the transcriptome sequencing data using the real-time (RT)-PCR (Fig. 6b).

### Putative roles of AS for drought stress regulation in potato

**Fig. 7:**
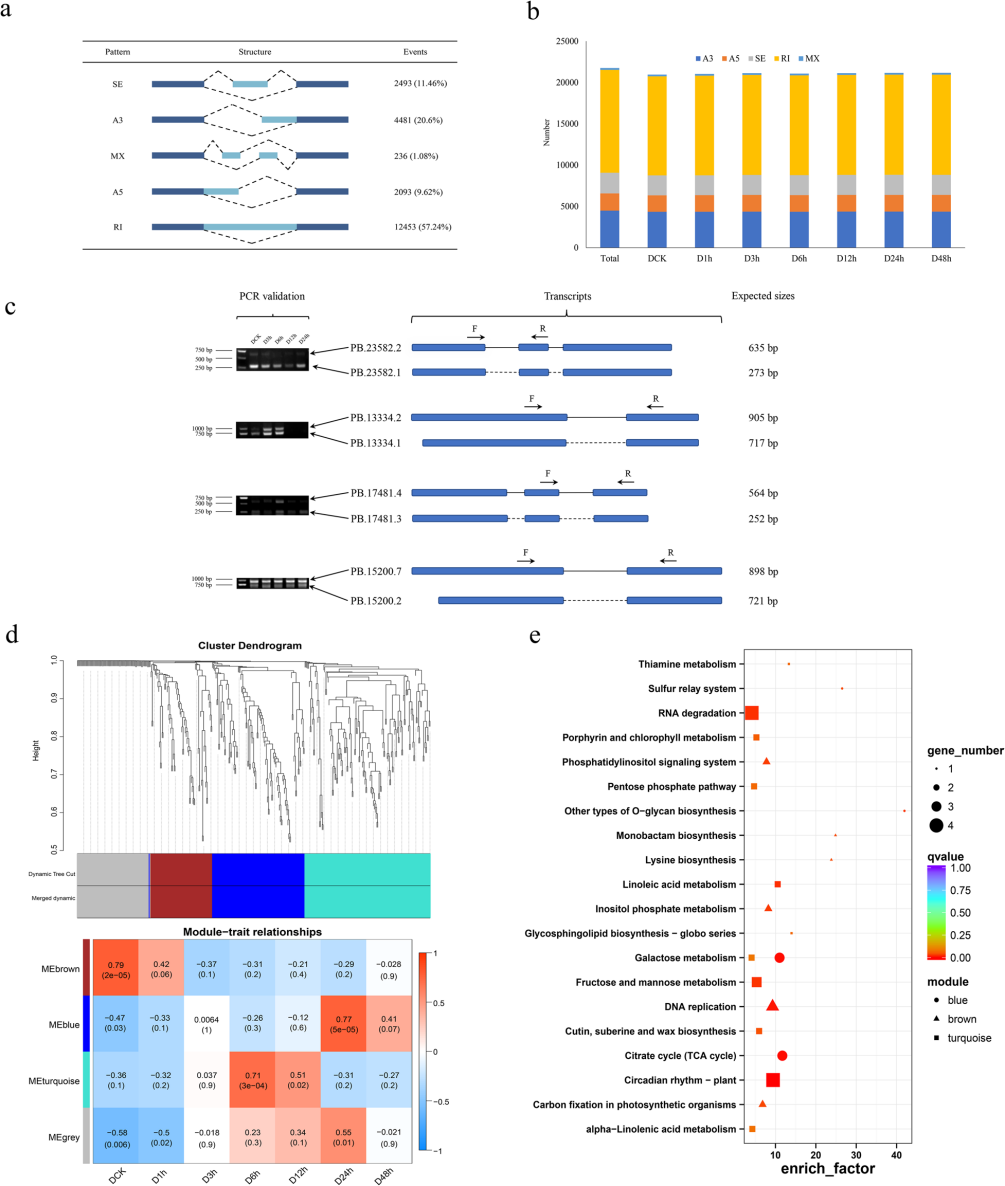
Expression profiles and functional analysis of alternative splicing (AS) events. **a**. Classification of AS events. Diagrams show the following AS events: retained intron (RI), alternative 3′ splicing sites (A3), alternative 5′ splicing sites (A5), mutually exclusive exons (MX) and exon skipping (ES). The numbers of AS events. Filled boxes represent exons, introns are represented by black lines. **b**. The number of differentially expressed AS events in DCK, D1h, D3h, D6h, D12h, D24h and D48h. **c**. Reverse-transcription (RT)-PCR validation of AS events. The RT-PCR validation of AS events for four genes, all of them are intron retention. Gel bands in each figure show DNA markers and PCR results in five samples (DCK, D3h, D6h, D12h and D24h). The transcript structure of each isoform is shown in the right panel. Exons are represented by blue filled boxes, introns are represented by lines, and the dotted line indicates that there is no intron corresponding to the first isoform in the second isoform of the same gene. Primers are designed to span the splicing events. PCR primers (F, forward and R, reverse) are shown on the first isoform of each gene. The length of each expected PCR product is shown after the transcript structure. **d**. WGCNA analysis of genetic modules relating to each sample. **e**. Enriched KEGG pathway of AS events involved in each module

AS plays critical roles in abiotic stress response by increasing genetic diversity in plants [36, 42]. However, little information about AS involved in drought stress response has been obtained in potato. In this study, 21,756 AS events were categorized into five patterns, including 12,453 retained intron (RI), 2493 skipping exon (SE), 236 mutually exclusive exons (MX), 4481 alternative 3′ splicing sites (A3), and 2093 alternative 5′ splicing sites (A5) using Isoform sequencing technology, which yields long reads without assembly (Fig. 7a). There is similar number of AS events for control and drought treatments, among these AS events, RI events predominated, accounting for 57.2%, followed by A3, SE, A5 and MX (Fig. 7b), which was consistent with findings of previous reports in other plant species [83]. Four random selected genes with AS events were successfully validated by reverse transcription (RT)-PCR (Fig. 7c).

To identify the co-expression network of genes involved in AS events, the WGCNA package was used and three significant modules were identified (brown, blue and turquoise). The DCK sample correlated to the brown module (*r* = 0.79, *P* = 2e^− 5^), whereas the turquoise (*r* = 0.71, *P* = 3e^− 4^) and blue (*r* = 0.77, *P* = 5e^− 5^) modules were significantly correlated with samples after 6 and 24 h treatments, respectively (Fig. 7d). KEGG enrichment of the significant modules was also performed. Six, eleven and four pathways were significantly enriched in brown, turquoise and blue modules. The galactose metabolism (ko00052) was significantly enriched in turquoise and blue modules, which were treated with PEG-6000 after 6 and 24 h, respectively (Fig. 7e).

### Putative roles of lncRNA involved in drought stress regulation in potato

**Fig. 8:**
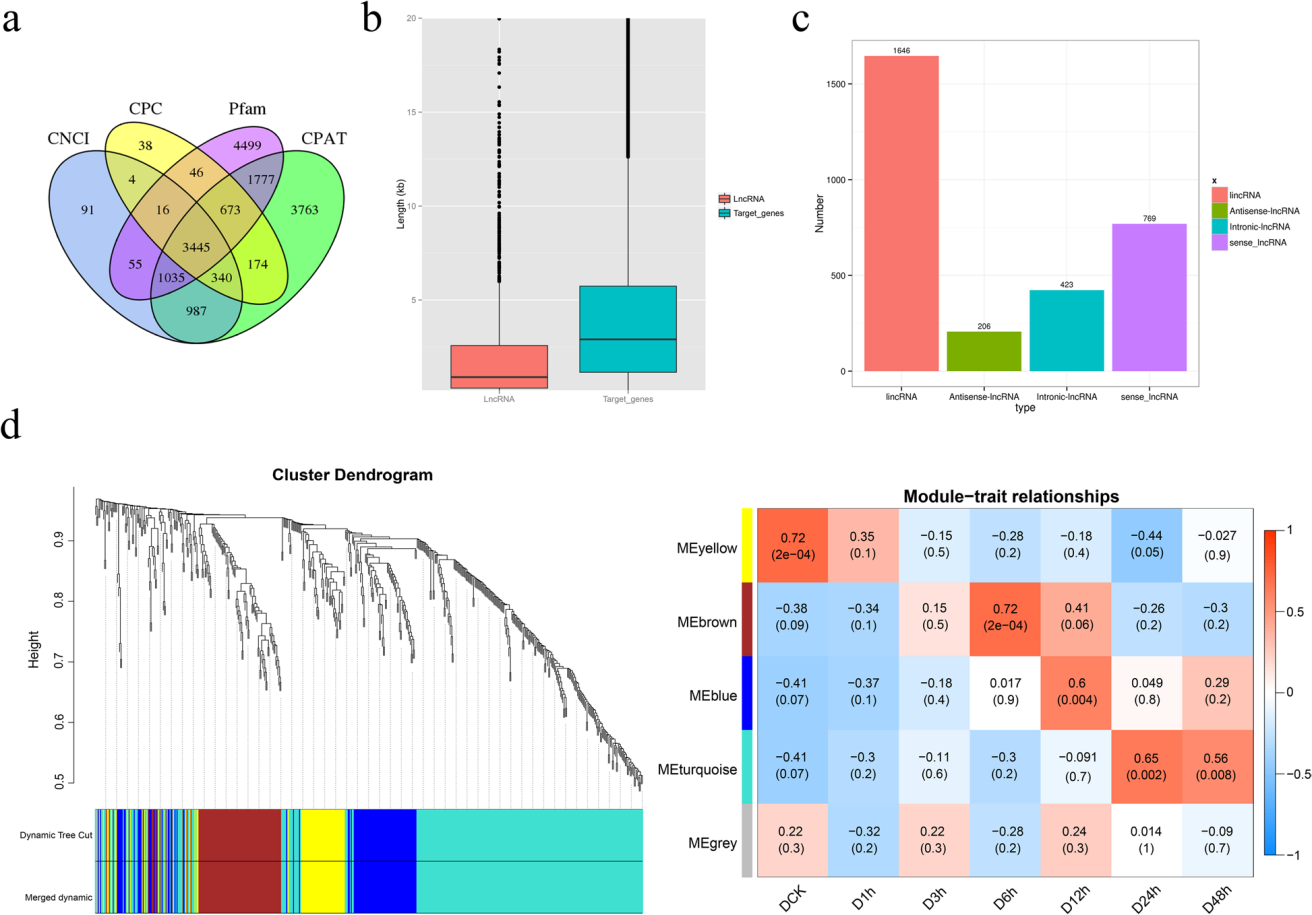
Expression profiles and functional analysis of lncRNA. **a**. Venn diagram of long non-coding RNA (lncRNA) prediction using four databases. **b**. LncRNA length and targeted genes. **c**. Distribution of different lncRNAs. **d**. WGCNA analysis of genetic modules relating to each sample

**Fig. 9:**
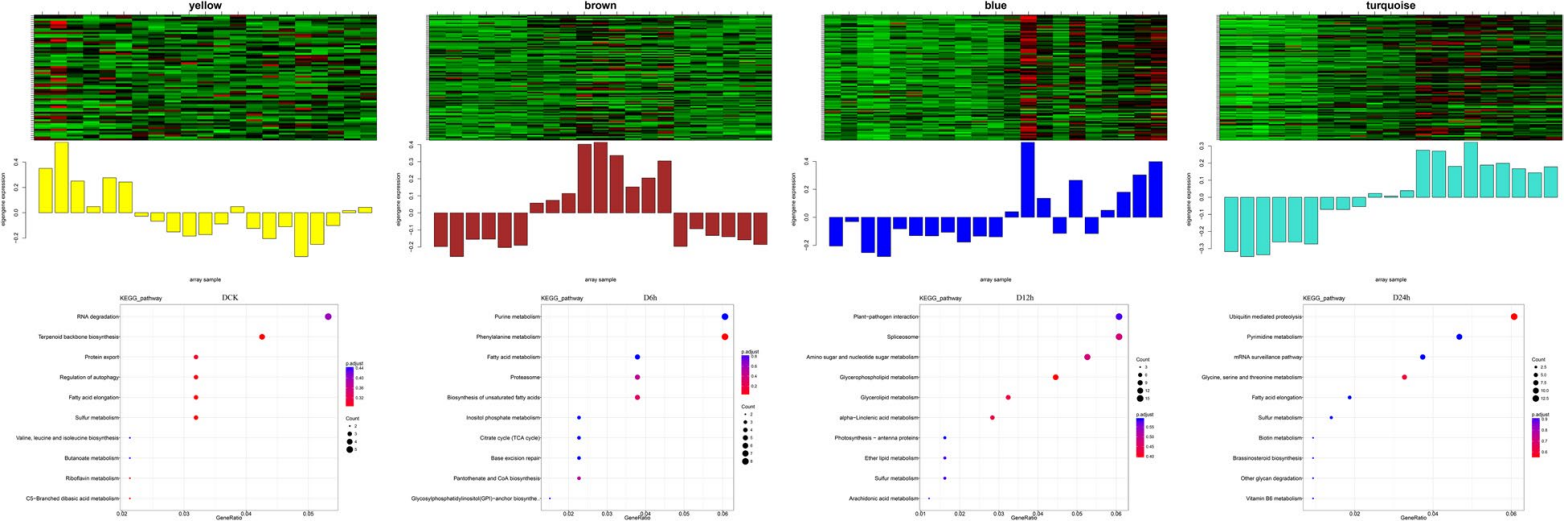
KEGG enrichment among the WGCNA modules most significantly correlated with lncRNA target genes. The figure shows the heat map and KEGG enrichment of four samples (DCK, D6h, D12h, D24h) [30–32]

LncRNA is a major component in transcriptome sequencing, and plays important roles in the regulation process after transcription [34, 39, 50, 58]. However, few information about lncRNA in potato has been obtained. In this study, 3345 lncRNAs with various lengths were identified using composite computational algorithm based on the PacBio sequencing data (Fig. 8a, b). More than 54% of lncRNAs were located in the intergenic region while less than 7% were located in the antisense region according to reference annotations (Fig. 8c). To identify the co-expression network of target genes of lncRNAs, the WGCNA package was used and yellow, brown, blue and turquoise modules were significantly enriched (Fig. 8d, Supplementary Table S5). The lncRNA target genes of KEGG analysis indicated that terpenoid backbone biosynthesis (ko00900) and phenylalanine metabolism (ko00360) pathway were most enriched in DCK and D6h; Glycerophospholipid metabolism (ko00564) and ubiquitin mediated proteolysis (ko04120) pathway were most enriched in D12h and D24h, respectively (Fig. 9).

### Alternative polyadenylation analysis

**Fig. 10:**
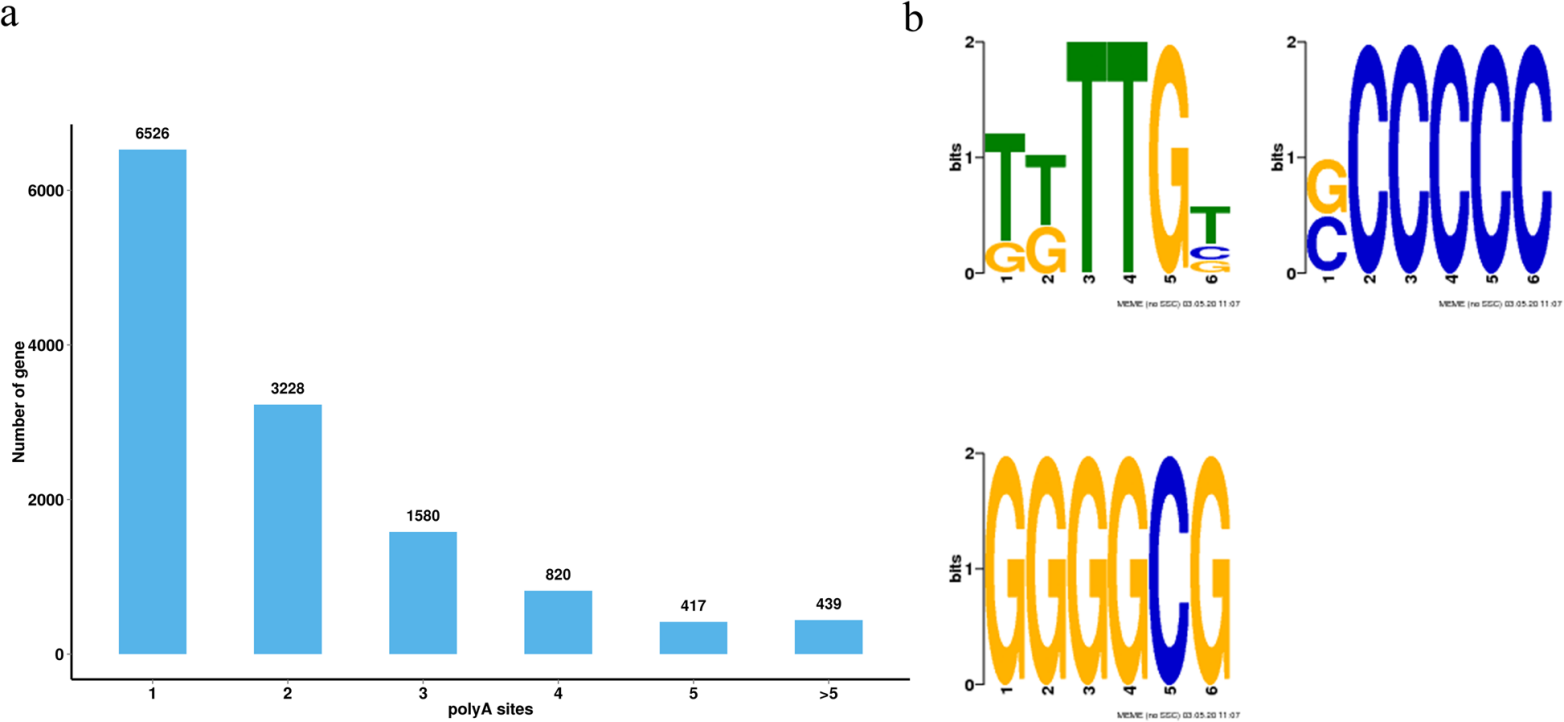
Alternative polyadenylation analysis. **a**. Distribution of the number of poly (A) sites per gene model. **b**. MEME analysis identified poly (A) signals in transcripts

In eukaryotes, polyadenylation is an important transcriptional modification in mRNAs [15, 61]. Alternative polyadenylation can easily increase the transcripts variety and regulate gene expression by producing different transcripts [18, 60, 62]. The iso-seq data allowed us to detect polyadenylation sites in potato using the program TAPIS [1]. In this study, 26,153 poly (A) sites from 13,010 genes detected in the Iso-Seq data were identified (Supplementary Table S6). On average, 2.01 poly (A) sites per gene were found and 856 genes had at least five poly (A) sites (Fig. 10a). In addition, a clear nucleotide bias of the flanking region of all poly (A) sites was found. To confirm this phenomenon, we investigated the motifs of 50 nucleotides upstream of predominant sites using MEME analysis. Furthermore, three conserved polyadenylation signals, i.e. TTTTGT, GCCCCC and GGGGCG were overrepresented in upstream of poly (A) cleavage sites (Fig. 10b).

## Discussion

Potato has important economic value, while they are vulnerable to drought stress. Therefore, it is important to understand the drought resistance mechanism of potatoes. Transcriptome research is one of the indispensable tools for understanding life processes. However, RNA-Seq technology based on the second-generation high-throughput sequencing platform often cannot accurately obtain or assemble complete transcripts, and cannot identify Isoform, homologous genes. Full-length transcriptome sequencing based on PacBio SMRT does not need to interrupt RNA fragments, and directly reverse transcribes the full-length cDNA, which enables the feasible determination of the whole transcriptome at the isoform level [1]. Previous study accelerated cloning of a potato late blight-resistance gene using RenSeq and SMRT sequencing [77]. Recently, a chromosome-scale long-read reference assembly has been constructed [55]. So far, a variety of potato species such as *S. chacoense* “M6” and *S. pinnatisectum*-derived somatic hybrid have been sequenced using Illumina platform [38, 71], and Kyriakidou et al. cultivated potato taxa using Illumina and long-read (PacBio) technologies [35]. In our research, NGS and SMRT sequencing technologies were used to study the transcription profile of potato in response to drought stress. The leaves of young plants of the variety “Désirée” after drought stress were used for NGS and SMRT sequencing at six time points from 0 to 48 hours. Statistical analysis of sequencing data revealed the important roles of DEGs, alternative splicing, LncRNA and APA in potato under drought stress.

### DEGs involved in drought stress response

In this study, SMRT and NGS technologies were combined to conduct a global survey of drought-treatment potato transcriptome. With the intensification of drought stress, the number of DEGs increased. The number reached the highest at 24 h after drought stress treatment (Fig. S1 and Supplementary Table S2). which was consistent with the findings previously reported in potato [10]. Plant hormones, such abscisic acid (ABA), auxin (IAA), and cytokinin (CK), play vital roles in abiotic stresses [72]. Transcription factors and plant hormone-related genes in differentially expressed genes were analyzed, and found that drought stress-related transcription factors such as bZIP and plant hormone-related genes such as ABA-related genes may played a dominant role. The functional annotation of differentially expressed genes confirmed their important role in drought stress, and 29 DEGs we verified using real-time (RT)-PCR (Supplementary Table S1 and Fig. 6b). Moreover, the WGCNA screening and KEGG enrichment analysis of drought stress response genes suggested that plant-pathogen interactions and glutathione metabolism pathways were enriched in the early stage of drought stress, and galactose metabolism pathways were enriched in at least two treatment samples, which indicated these pathways play an important role in the potato drought response process. Previous researches reported that galactose is involved in response to drought, high salt and low temperature tolerance [70], and transgenic studies demonstrated that glutathione helped *Tortula ruralis* adapt to rehydration following rapid desiccation [14]. It was clear that the number of genes down regulated was greater that the upregulated which means that the plant stops regular active metabolism as main mechanism of response to the drought stress.

### AS events involved in drought stress response

One of the advantages of PacBio sequencing is its ability to detect AS events. (Wang, [75, 87]). For different biological responses, AS is an effective mechanism to increase the complexity and flexibility of the transcriptome [41]. Previous studies have confirmed that AS events play critical roles in potato growth and abiotic stress response [43, 52]. AS events identified during the potato drought stress response process were analyzed in this study. The proportion of different AS events was consistent with the findings previously reported in other plant species [80, 83]. Our experiment verified the AS events detected above, and the results showed that the size of the gel band fragments was consistent with the splice isoforms identified from the Iso-Seq data (Supplementary Table S1 and Fig. 7c) and the functional annotations of these genes also indicated that they were involved in drought stress response. In summary, we have verified that the four randomly selected genes undergo alternative splicing under drought stress, and their functional annotations are also related to plant stress tolerance. Our experiments results and sequencing data indicated that potatoes may increase their protein diversity under drought stress through alternative splicing, and thereby improve their environmental adaptability.

### LncRNA plays an important role in drought stress response

As a type of non-coding RNA, lncRNA act as regulators in a series of biological processes [26]. Previous research suggested that lncRNA has potential functions in potato defense response and development [34, 56]. In this study, a total of 3445 lncRNAs of different lengths were identified through four methods: CPC analysis, CNCI analysis, pfam protein domain analysis, and CPAT analysis (Fig. 8a and b). The WGCNA and KEGG analysis results of lncRNA target genes show the term ‘phenylalanine metabolism’ was enriched in the brown module, indicating that lncRNAs respond to drought stress by targeting genes related to amino acid metabolic pathways (Fig. 9), it also provided additional effective candidate genes for future functional characterization.

### APA is essential for drought resistance of potato

Polyadenylation refers to the covalent linkage of polyadenylic acid to messenger RNA (mRNA) molecules. In the process of protein biosynthesis, this is part of the way to produce mature mRNA ready for translation. In eukaryotes, polyadenylation is a mechanism that interrupts mRNA molecules at their 3’ends. The polyadenylic acid tail (or poly A tail) protects mRNA from exonuclease attack, and is very important for the termination of transcription, export of mRNA from the nucleus, and translation. A comprehensive genome-wide APA map draft consisting of 26,153 poly (A) sites from 13,030 genes was constructed in this study (Supplementary Table S6). The nucleotide bias was evaluated and obvious nucleotide bias in the flanking regions of all poly (A) sites were found (Fig. 9b). These findings are consistent with those of previous studies in *Brassica napus* [84]. Two conserved polyadenylation motifs in *B. napus* were identified, while three conserved polyadenylation motifs in potato were identified, indicating that the polyadenylation motifs are species-specific [40]. Previous studies have shown that alternative polyadenylation of RNA is critical for gene function by increasing the complexity of the transcriptome and regulating gene expression [79].

## Supplementary Information

**Additional file 1: Supplementary Fig. S1.** Volcano plots of expression comparisons in different drought treatment time. **Supplementary Fig. S2.** KEGG enrichment among the WGCNA modules most significantly correlated with drought-treated samples. The figure shows the heat map and KEGG enrichment of four drought-treated samples (D1h, D6h, D24h, D48h) [30–32]. **Supplementary Table S1**. Summary of primers used in this study. **Supplementary Table S2**. The number of differentially expressed genes. **Supplementary Table S3**. FPKM, fold changes, FDR and functional annotation of DEGs. **Supplementary Table S4**. Intersection data of DEGs in six samples. **Supplementary Table S5**. Prediction of lncRNA target genes based on co-expression. **Supplementary Table S6**. Statistics and localization of genes with poly (A) sites.

**Additional file 2: Fig. 7**c. Original figures of gels.

## Data Availability

The datasets generated and analyzed during the current study are available in the NCBI Sequence Read Archive repository under Bioproject PRJNA728834 (https://www.ncbi.nlm.nih.gov/Traces/study/?acc=PRJNA728834).
