# Supplementary figures and images for: Construction of drought stress regulation networks in potato based on SMRT and RNA sequencing data
Source: BMC Plant Biol. 2022 Aug 1;22:381. doi: 10.1186/s12870-022-03758-8 (PMC9341072; doi:10.1186/s12870-022-03758-8)

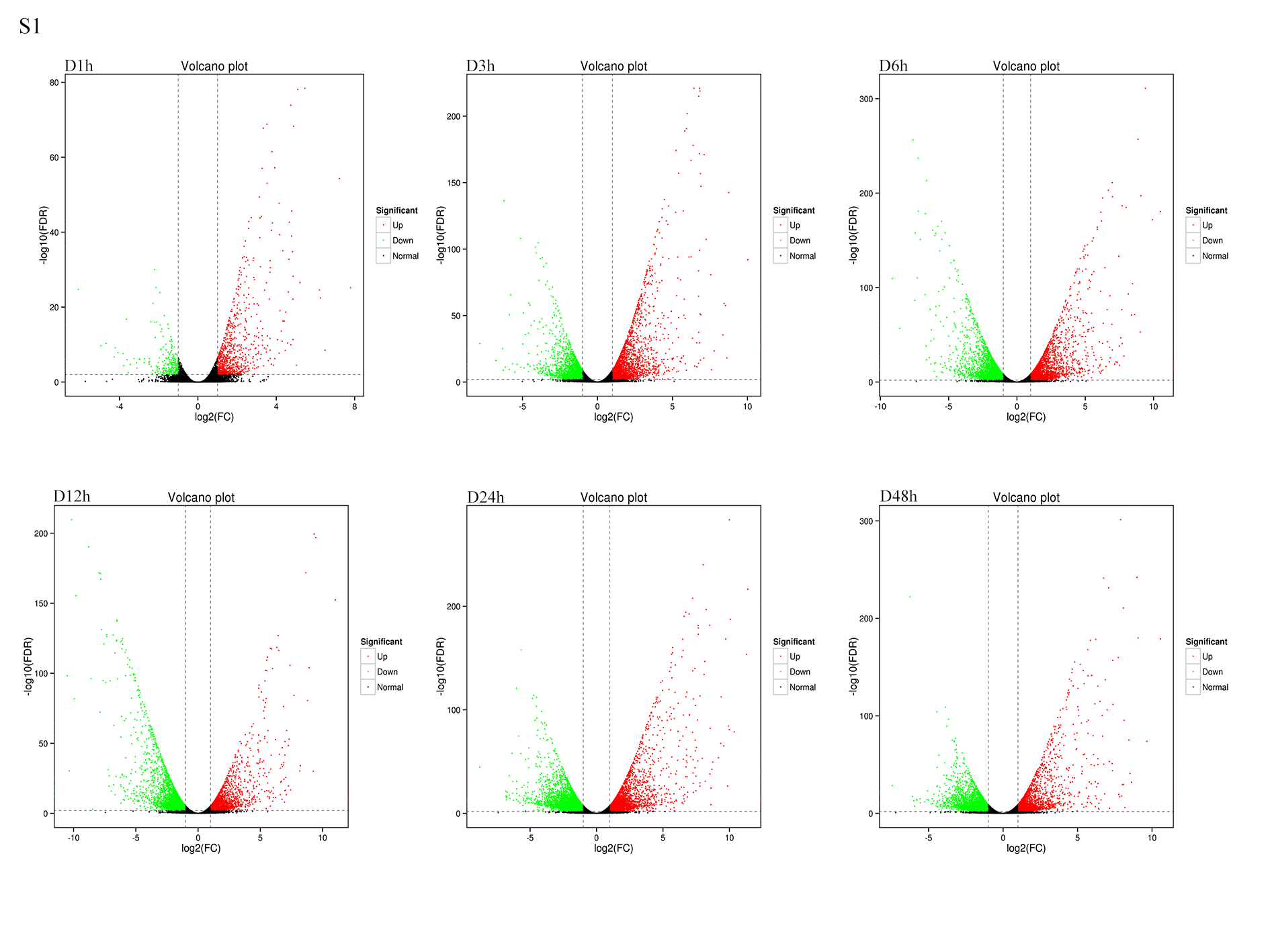

Supplement: Supplementary file 1 — Additional file 1: Supplementary Fig. S1. Volcano plots of expression comparisons in different drought treatment time. Supplementary Fig. S2. KEGG enrichment among the WGCNA modules most significantly correlated with drought-treated samples. The figure shows the heat map and KEGG enrichment of four drought-treated samples (D1h, D6h, D24h, D48h) [30–32]. Supplementary Table S1. Summary of primers used in this study. Supplementary Table S2. The number of differentially expressed genes. Supplementary Table S3. FPKM, fold changes, FDR and functional annotation of DEGs. Supplementary Table S4. Intersection data of DEGs in six samples. Supplementary Table S5. Prediction of lncRNA target genes based on co-expression. Supplementary Table S6. Statistics and localization of genes with poly (A) sites. [file 12870_2022_3758_MOESM1_ESM.zip › Fig. S1.tif]

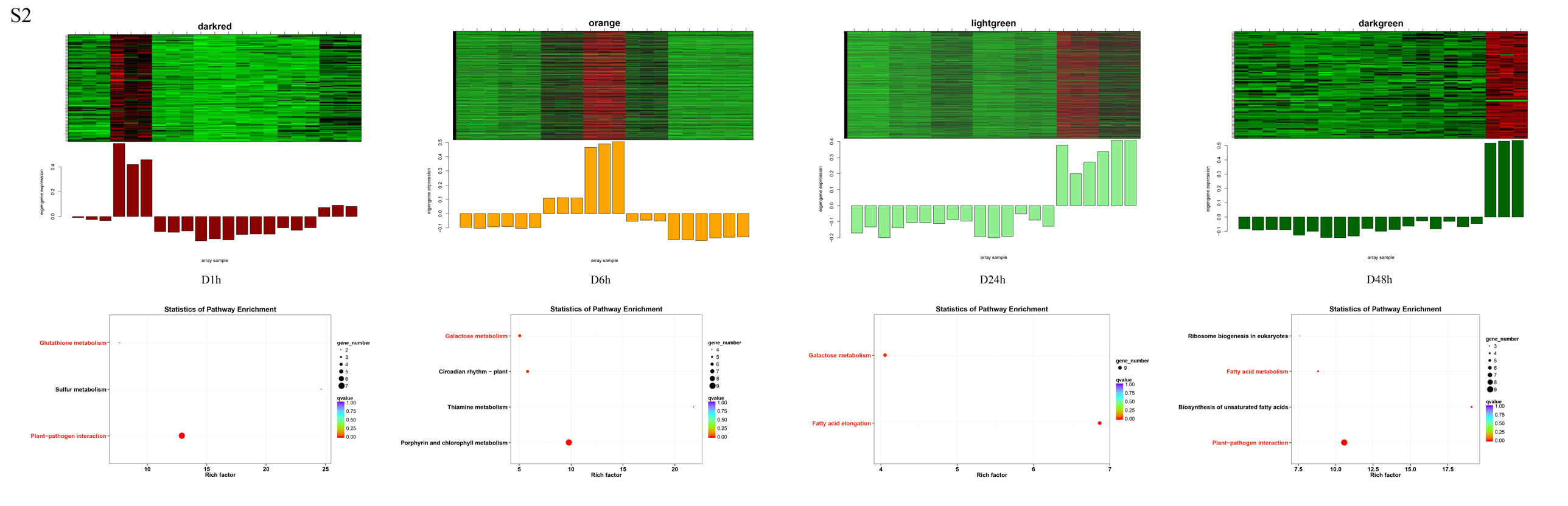

Supplement: Supplementary file 1 — Additional file 1: Supplementary Fig. S1. Volcano plots of expression comparisons in different drought treatment time. Supplementary Fig. S2. KEGG enrichment among the WGCNA modules most significantly correlated with drought-treated samples. The figure shows the heat map and KEGG enrichment of four drought-treated samples (D1h, D6h, D24h, D48h) [30–32]. Supplementary Table S1. Summary of primers used in this study. Supplementary Table S2. The number of differentially expressed genes. Supplementary Table S3. FPKM, fold changes, FDR and functional annotation of DEGs. Supplementary Table S4. Intersection data of DEGs in six samples. Supplementary Table S5. Prediction of lncRNA target genes based on co-expression. Supplementary Table S6. Statistics and localization of genes with poly (A) sites. [file 12870_2022_3758_MOESM1_ESM.zip › Fig. S2.tif]

Fig. 7c: original figures of gels

PB.23582


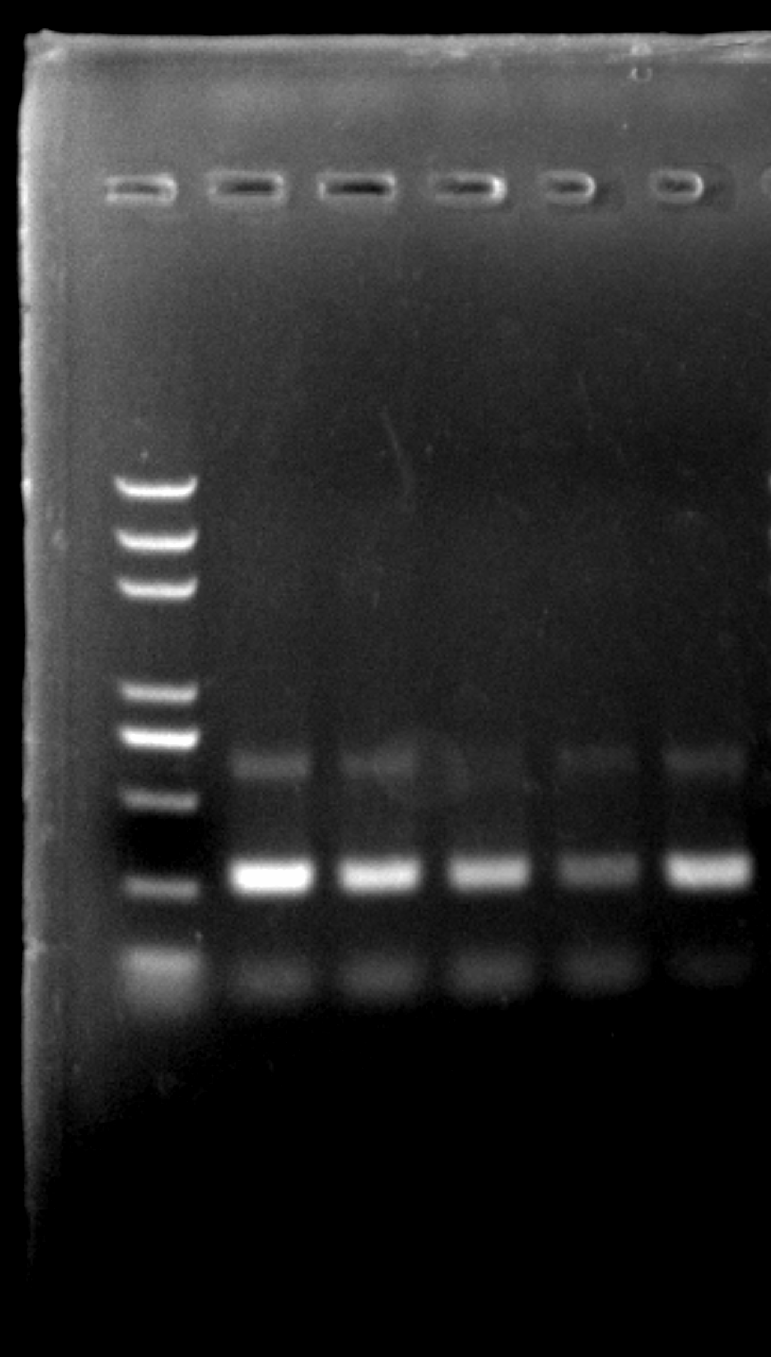


PB.13334


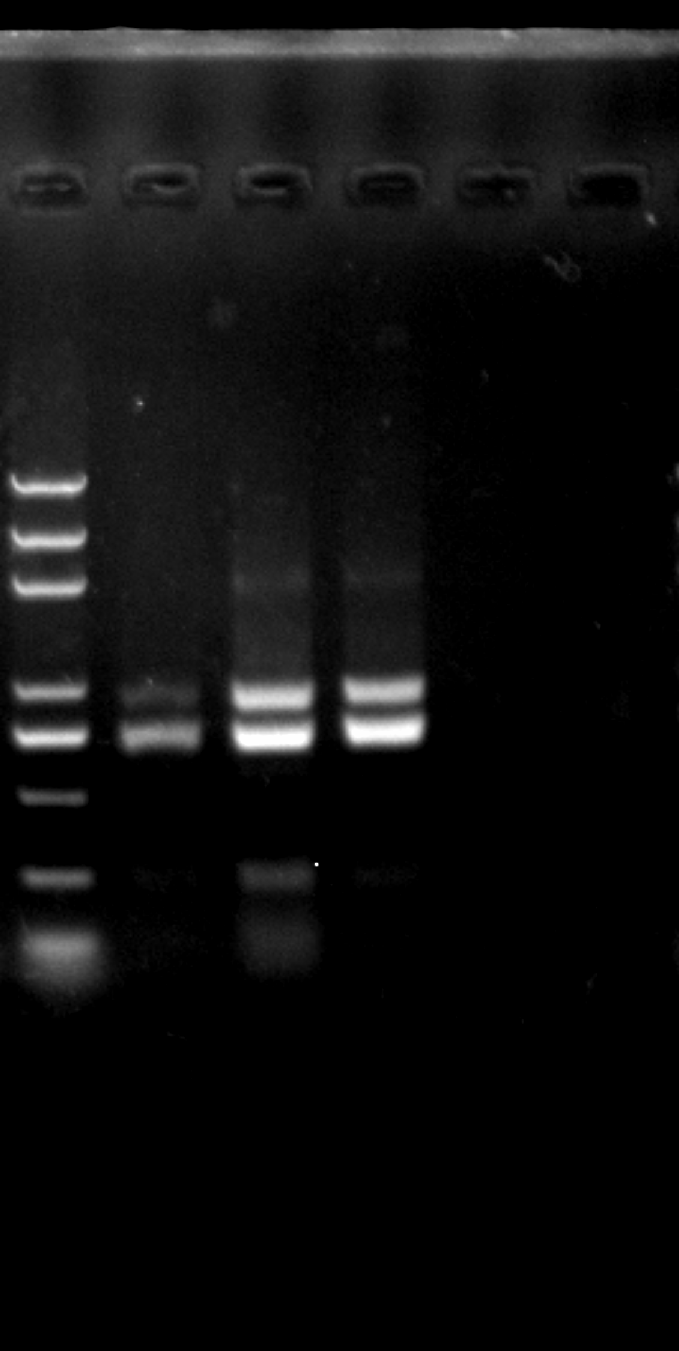


PB.17481


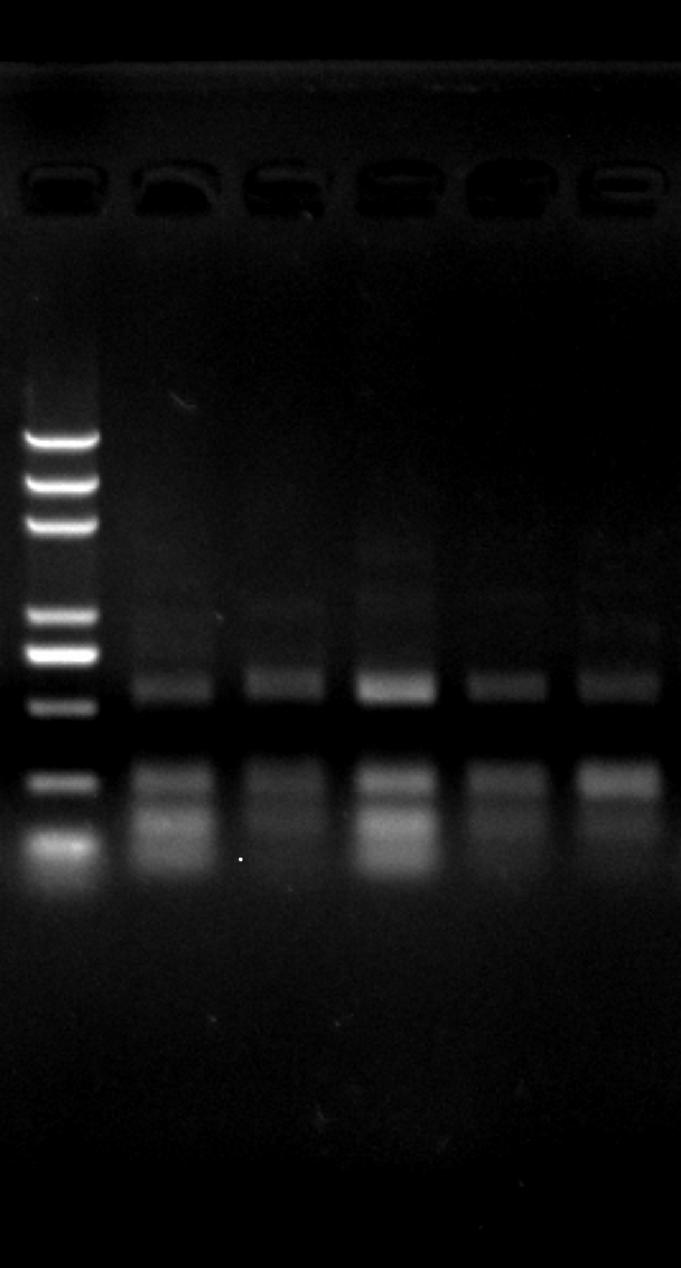


PB.15200


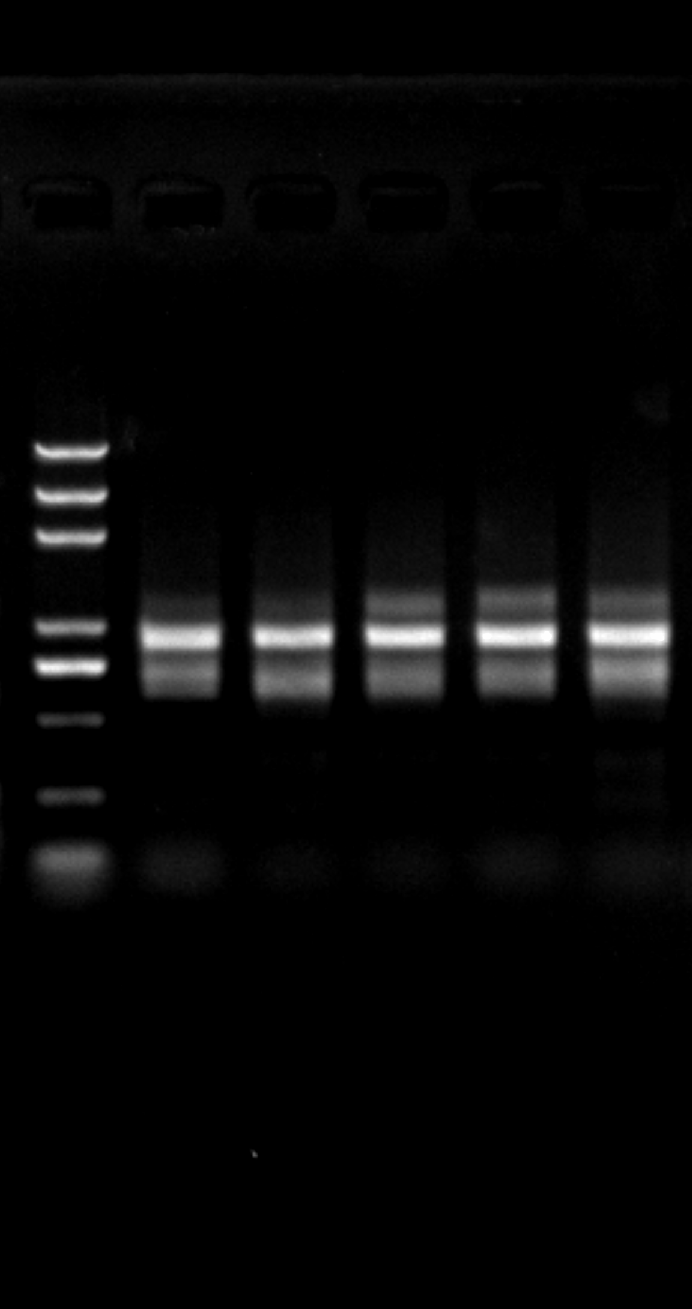

Supplement: Supplementary file 2 — Additional file 2: Fig. 7c. Original figures of gels. [file 12870_2022_3758_MOESM2_ESM.docx]
